# Toward Fully Automated Inspection of Critical Assets Supported by Autonomous Mobile Robots, Vision Sensors, and Artificial Intelligence

**DOI:** 10.3390/s24123721

**Published:** 2024-06-07

**Authors:** Javier Sanchez-Cubillo, Javier Del Ser, José Luis Martin

**Affiliations:** 1 ZeniaLabs Automation Intelligence, 48160 Derio, Bizkaia, Spain; jscubillo@zenialabs.com; 2 TECNALIA, Basque Research & Technology Alliance (BRTA), 48160 Derio, Bizkaia, Spain; joseluis.martin@ehu.eus; 3 Bilbao School of Engineering, University of the Basque Country (UPV/EHU), 48013 Bilbao, Bizkaia, Spain

**Keywords:** autonomous mobile robot, autonomous guided vehicles, artificial intelligence, object detection

## Abstract

Robotic inspection is advancing in performance capabilities and is now being considered for industrial applications beyond laboratory experiments. As industries increasingly rely on complex machinery, pipelines, and structures, the need for precise and reliable inspection methods becomes paramount to ensure operational integrity and mitigate risks. AI-assisted autonomous mobile robots offer the potential to automate inspection processes, reduce human error, and provide real-time insights into asset conditions. A primary concern is the necessity to validate the performance of these systems under real-world conditions. While laboratory tests and simulations can provide valuable insights, the true efficacy of AI algorithms and robotic platforms can only be determined through rigorous field testing and validation. This paper aligns with this need by evaluating the performance of one-stage models for object detection in tasks that support and enhance the perception capabilities of autonomous mobile robots. The evaluation addresses both the execution of assigned tasks and the robot’s own navigation. Our benchmark of classification models for robotic inspection considers three real-world transportation and logistics use cases, as well as several generations of the well-known YOLO architecture. The performance results from field tests using real robotic devices equipped with such object detection capabilities are promising, and expose the enormous potential and actionability of autonomous robotic systems for fully automated inspection and maintenance in open-world settings.

## 1. Introduction

Autonomous mobile robots (AMRs) and autonomous guided vehicles (AGVs) are becoming increasingly important in the industrial sector, emerging as relevant parts of the robotic devices that support modern production and inspection processes within the Industry 5.0 paradigm. A few years ago, it was common to see factory personnel manually moving parts between assembly lines for robotic manipulators. Nowadays, it is more typical to find conveyor belts or mobile robots transporting these parts between different factory areas. The latter option offers significantly more flexibility in managing loading and unloading zones and allows for the dynamic configuration of various industrial scenarios on demand.

Both systems can be considered mobile robots and can self-navigate without human oversight. The main difference between them lies in their ability to navigate without external guidance. AMRs are intended to make decisions and change the navigation path if needed. Conversely, AGVs are normally confined to tracks or predefined paths. Both robotic platforms are usually equipped with numerous sensors and must have the ability to stop their operation in emergencies, either due to dangerous circumstances or risks to human personnel. With the wide array of advanced sensors installed on these robotic platforms, AMRs and AGVs can be more efficient and faster than humans in emergency-stopping tasks, as humans are susceptible to distractions and have slower reaction times. Indeed, these superior sensing and perception capabilities make AMR particularly suitable for automated mobility and asset inspection, far beyond just moving loads in warehouses as discussed previously.

However, there are certain actions and decisions of mobile robots, especially in unknown and unstructured environments, where conventional sensors that measure physical quantities face significant limitations in identifying objects within a scene. Traditional sensors are highly efficient at measuring distances, speeds, accelerations, temperatures, and frequencies. Along with signal processing and filtering algorithms, they provide a reliable and comprehensive perceptual representation of the environment. Unfortunately, this combination of sensors and conventional signal processing usually proves very ineffective in complex situations, such as those arising from the inspection of critical assets under environmentally complex circumstances. In such situations, a cognitive understanding of those magnitudes, measured by the sensors installed in the AMR, is required. Signal processing and filtering easily find obstacles based on the signals captured by the sensors. Unfortunately, they lack the perceptual capabilities to detect and classify between different objects. There are situations in which it is necessary to discriminate objects from each other, locating them in a specific space, even when they are partially occluded. This is an obvious and relatively simple task for the human eye and is crucial for decision-making in complex environments. Among them, in this work, we focus on the inspection of critical assets, where the detection, identification, and characterization of objects on the asset at hand is of utmost importance to ensure its safe operation.

With the progressive maturity of artificial intelligence (particularly machine learning, ML), the aforementioned limitations of AMRs in environmental perception are starting to dissolve, opening up new perspectives that surpass the capabilities of traditional vision sensors and signal processing alone. At their current level of maturity, ML models are gradually being incorporated into industrial products, typically consumer goods where AI adds value to the detection, discrimination, localization, and classification capabilities of certain devices. However, this technology does not determine actionable decisions that entail dangerous operations or risks in the task.

In this work, we explore the potential of ML to assist AMRs in fully automated inspection and maintenance tasks. Our overarching goal is to assess the performance of established models for object detection across three different real-world use cases for automated inspection, each with its unique characteristics. Given their mobility and navigation capabilities, the robotic platforms presented in this research serve both as AGVs and AMRs, depending on the target situation and the characteristics of the terrains where they operate:

- On the one hand, the first use case involves a fully robotized AMR platform developed for the rail sector, with the ability to freely navigate between railway tracks without stopping rail traffic. The AMR platform is designed for the automated inspection and detection of weed coverage, providing an alternative to the use of glyphosate by potentially allowing for their elimination by the robot.
- On the other hand, the other two use cases involve AGVs from two different forklift platforms, each with varying load capacities. These vehicles, originally operated by human drivers, have been fully robotized by removing the driver from the driving task. They are equipped to navigate a sensorized and predefined path within a warehouse while transporting a payload. They are also able to navigate freely, for example, in a port environment or within a truck without the help of infrastructure sensors. The two use cases focused on in our study are the automated inspection of maritime twenty-foot equivalent unit (TEU) containers and the automated loading of goods directly into a truck using the mobile robot.

Our experimental results consider the three novel robotic AMR- and AGV-based applications mentioned above, which perform inspection and loading tasks in complex environments. Using real data collected from these Use Cases By the robotic platforms, we conduct a performance benchmark of one-stage ML models for object detection, employing established evaluation protocols and performance scores. To perform the assigned tasks, the three robots must possess extraordinary detection and discrimination capabilities to perceive the environment and manipulate objects, which cannot be achieved with conventional computer vision and require the inclusion of AI. Based on the results, we conclude that the synergy between AMR/AGV and object detection is promising, paving the way for new use cases that leverage the autonomy and intelligence embodied by this technological crossroads.

**Contribution:** The contribution to the state of the art made by our research herein lies in providing an in-depth analysis of the real benefits of artificial intelligence on industrial robotic platforms. Inspection robots work in complex, unpredictable, and very unstructured environments, so for the sake of meeting their objectives, our research aims to provide metrics on the performance achieved with AI inclusion that cannot be achieved otherwise. We also develop and provide the frameworks that enable the inclusion of these models effectively in the proposed rail, port, and logistics sectors. These sectors are known for being very conservative regarding the adoption of new and emerging technologies. However, the results of our field tests in real environments for each Use Case Demonstrate that adopting AI for robotic inspection is an actionable and practical approach for fully automating this task, and it outlines a promising future for applications leveraging AI-assisted robotics.   

The rest of the manuscript is structured as follows: we first briefly review the state of the art in visual perception in AGV/AMR (Section 2), followed by a description of the robotic design of these platforms within the three use cases under consideration (Section 3). We then describe the experimental setup in Section 4, followed by a discussion on the obtained results and the insights drawn from the field tests conducted for the three use cases (Section 5). Finally, Section 6 concludes the paper with a summary of the main conclusions drawn from this research, together with a glimpse at future research.

## 2. Related Work

Before proceeding with the description of the AMR and AGV robotic platforms used in our research, we first provide some background on these robotic technologies themselves (Section 2.1 and Section 2.2), followed by visual perception in AMR platforms (Section 2.3).

### 2.1. Mobile Robotic Inspection: Architectures and Usages

Robots for inspection, both in industry and research, feature a wide range of architectures, traction, and navigation [1]. The differences stem from the specific tasks for which they are designed. There are multiple architectures for inspection robots depending on the inspection site, and these can be categorized based on their architecture. Here are several examples of industrial inspection robots based on their architecture:

1. Mobile robots with fully rotational wheels, which are commonly found in both industry and research. These wheels can turn a full 360 degrees, allowing the robot to rotate on its axis, which is highly valuable for inspections in areas with very limited space for turning.
2. Robots on rail tracks, such as those used for rail inspection, similar to the one examined in this research, and in other configurations where operations occur while trains are stopped.
3. Robots on caterpillar tracks.
4. Four-legged robots, emulating animals.
5. Omni-wheel-based robots, where the wheel can move in any direction.
6. Submarine robots.
7. Flying drone robots.
8. Tethered and snake robots for pipe inspections.

Despite the large structural differences, the perception and handling systems of test instruments can be very similar in all types of robots, taking into account the different load capacities of each of them.

The tasks to be performed by robots in complex, unstructured environments, (e.g., outside controlled environments within a cell in a production warehouse), require advanced visual skills and dexterity through specialization, which—until recently—only humans could perform. To succeed in these tasks, it is necessary to have the ability to differentiate between nominal events and anomalous situations and to have the capacity to resolve each of these situations in a satisfactory manner without putting the product, people, or the work environment itself, at risk [2].

This differentiation is becoming essential in a highly automated and digitized industrial environment. However, the ability of robots to distinguish themselves increasingly relies on the continuous layering of ever more accurate, smaller, faster, and more economically viable sensor systems and automation for mainstream adoption. The suitability of using mobile robots seems optimal in situations where the automation, vision, inspection, or manipulation system must be directed toward the specific task, either because the object itself cannot be moved to where the robots are located (as in the case of manufacturing factories), or because of the human or economic cost that moving would entail. In work by Dudek et al. [3], the suitability of mobile robots is described following the characteristics of the environment where they are intended to operate:

1. An inhospitable environment, so deploying a human being is either very costly or very dangerous.
2. A remote environment, so that sending a human operator is too difficult or takes too long.
3. Extreme instances are domains that are completely inaccessible to humans, such as microscopic environments.
4. A task with a very demanding duty cycle or a very high fatigue factor.
5. A task that is highly disagreeable to a human.

The three first items (1–3) refer to environments where the allocation of human workforce is normally not possible, or not well suited, like the planetary exploration in the space sector, the deep maritime environment, extremely dangerous and inaccessible mining caves, or extremely dangerous operations for humans due to radioactivity or explosive danger. The two following items (4–5) refer to any industrial, sanitary, or rescuing operation, where the automated mobility of a robot can help reduce human injuries or severe accidents, improve productivity, and enable safe human remote intervention. In recent times, applications of AMR have also steered toward other challenging scenarios such as surveillance, disaster response, and consumer applications [4,5].

### 2.2. Autonomous Mobile Robots (AMRs) and Automated Guided Vehicles (AGVs)

Advances in AGV and AMR technology and their industrial applications have recently experienced an unprecedented boom, driven by the emergence of demand for this type of system in new scenarios previously limited to human labor. These mobile robots have been integrated into warehousing and logistics activities, using track-guided magnetic systems, optical sensors, and color bars as guidance technologies [6]. Zhang et al. [7] thoroughly reviewed the use of AGVs and AMRs for recognition and tracking in civil engineering, along with an analysis of the challenges and prospects in detection, construction, and disease repair. More recently, Patruno et al. [8] proposed an architectural taxonomy of several AMRs and AGVs is presented. Loganathan et al. [9] provided an exhaustive and systematic analysis of the strengths and shortcomings of navigation and path planning techniques for AMRs.

Depending on the environment where the robot will operate, a different motion drive system must be selected. Robots with drive systems that allow them to move in any direction, independent of their orientation, are called omnidirectional or *holonomic* robots. Omnidirectional robots are considerably easier to control than robots based on simple wheels because their motion is independent of their pose [3]; they use special types of wheels such as omnidirectional, mecanum, or fully 360º turnable wheels. However, they are usually intended for operation on flat floors. The demands of rough terrain, where our robots will operate in two of the three use cases, preclude the use of these types of wheels due to their motion characteristics and fragility, which would cause the robots to become stuck. Siegwart et al. [10] provided an excellent review of these techniques for wheeled robots in their book *Introduction to Autonomous Mobile Robots*.

Mobile robots, like the ones equipped with differential motion, can also turn on their axis, as is the case with the AMR designed for the rail use case (as detailed later). Other mobile robots with Ackermann [11] or simple wheels require more space for turns, as the turning maneuver always involves motion on their x-axis. This is, for example, the case with vehicles that use Ackermann steering. Selecting the right motion strategy is crucial, as it defines the navigation and path planning constraints that the control of the AMR will face [12].

In order to obtain a robust and reliable navigation system for mobile robots, it is essential to use the ROS/ROS2 [13] framework. All three robots presented here operate on the ROS2 framework running on a high-level processing computer based on the NVIDIA Jetson Orin architecture. They interact with other low-level industrial programmable logic controllers (PLCs), responsible for controlling all the input–output interfaces to the mechatronics and information buses, e.g., the controller area network (CAN), inter-integrated circuit (I2C), and serial peripheral interface (SPI) of the embedded electronics.

ROS, an open-source robotic operating system, supports most modern robotics research and commercial products. Its success stems from its focused design and enhanced capabilities tailored for product-grade robotic systems. ROS not only supports the generation of trajectories (global and local path planners) but also facilitates the integration of image processing libraries for environmental visual perception. This combination provides a robust programming framework for determining the optimal actions of the robot at all times.

### 2.3. Visual Perception in Autonomous Mobile Robots

The space sector is one of the most important pioneering sectors in the use of navigation and perception in mobile robots [14]. Research aimed at designing rovers for planetary exploration has excelled in providing autonomous robots for environments inaccessible to humans. It has allowed us to gain experience and learn from the perception systems required for route planning and navigation before such sensors were available to the general public. In the 1990s, sensor fusion techniques were primarily used for navigation and obstacle avoidance. These techniques enabled mobile robots to navigate safely in non-mapped and dynamic environments by perceiving their environment while deciding on actions based on those perceptions. Typical perception systems included sonar-based obstacle avoidance combined with vision-based pose determination [15] for detecting artificial or natural landmarks. The sensor data were integrated with two common obstacle avoidance methods: vector field histogram (VFH) and navigational templates (NaTs). The VFH obstacle avoidance algorithm utilizes a two-dimensional Cartesian grid, known as the histogram grid, to represent data from ultrasonic range sensors. Each cell in the histogram grid holds a certainty value that indicates the algorithm’s confidence in the presence of an obstacle at that location. Navigational templates (NaTs) combine high-level, qualitative guidance with low-level, quantitative control and are designed to address issues from VFH and other obstacle avoidance methods by influencing high-level decisions on which side of an obstacle the robot should pass [16,17].

Such systems and algorithms are still valid to date. However, a lot has changed since their inception. New sensors, algorithmic techniques, and powerful processing units for autonomous robotics have been developed in recent decades. Current AI-based processing systems can be considered successors to those early algorithms, now relying on smaller, more robust sets of sensors with enhanced accuracy for environmental perception. These include radar, 2D and 3D LiDAR, high-resolution stereoscopic and time-of-flight cameras, and inertial measurement units (IMUs). Also, new processing algorithms and libraries have entered the scene. Following the late 1990s, OpenCV (*Open Source Computer Vision Library*) became a key player in computer vision. It was initially created as part of an Intel Research initiative to advance CPU-intensive applications but has become a powerful open-source computer vision tool, provided and maintained by researchers and programmers for the community. The library is continuously evolving and now offers today more than 2500 optimized computer vision algorithms [18].

The use of software libraries like OpenCV [18], *Sci-kit Image* [19], *PyTorch* [20], and *TensorFlow* [21], together with other proprietary software packages from Google, Azure, Amazon, and Microsoft have enabled and simplified the processing of data from new sensors. They are not only software pieces used for processing data from pictures, videos, and vision cameras, they also provide processing algorithms for the new sensors mentioned before, by delivering tools such as image filtering, camera calibration, structure-from-stereo/structure-from-motion algorithms, visual odometry, feature detectors for cameras (Hough, Harris, FAST, SURF, and SIFT), and processing of laser point clouds. Some of the algorithms used within this research are worth mentioning due to their importance in object detection and classification tasks, namely the SIFT (scale-invariant feature transform) and SURF (speeded-up robust features) algorithms. SIFT detects distinctive key points or features in an image, keeping it resilient to variations in object size, orientation, rotation, or affine transformations. SURF is another algorithm for key-point detection and image feature description that offers increased computational speed, which is useful for real-time applications. Both are computer vision algorithms included in the OpenCV package for detecting and describing key features in images. They are of key importance since they lay the foundation for the detection and extraction of intrinsic features in images, which can subsequently be put on top of the layers for more complex AI-based detection and classification stages. Based on these well-known computer-vision algorithms, the key elements of this research focus on the added value of combining conventional algorithms with new AI-based ones. Sharma et al. [22] provide an extensive comparison of diverse feature detectors and descriptors. The most recent versions of OpenCV include packages for Markov and Kalman filter localization, simultaneous localization and mapping (SLAM) in its extended Kalman filter, graph-based SLAM, or particle-filter versions, and the latest monocular visual SLAM. Moreover, the OpenCV package supports the use of graph-search algorithms for path planning, such as breadth-first, depth-firs, Dijkstra, A*, D*, and rapidly exploring random trees, which are useful for navigation purposes [10].

In general, AGV and AMR recognition and tracking technology involve self-positioning, environmental perception, map construction, and path planning among the required abilities of the robots [7]. Apart from the ability to capture and process intrinsic characteristics of environmental images based on chromatic and morphological features as can be obtained with the described algorithms, the robots in our study require complex discrimination, detection, and classification of objects. Similar to how Blubaugh et al. [23] analyze the need for a mobile robot or rover to extract information from images, and recognize objects by their patterns and features to navigate the environment while avoiding obstacles, our robots require precise information about the environment and the scenarios in which they operate. One strict requirement is that they avoid collisions, as the objects in the infrastructure are customer-owned and sometimes operationally critical, and must be kept intact at all times.

Today, a new paradigm is opening up in the field of perception for mobile robotics through the use of artificial intelligence and deep learning techniques for visual detection. In their review, Cebollada et al. [24] showed how a variety of computer vision, AI, and deep learning tools are currently employed in mobile robotics. The use of deep learning techniques for inspection activities in industrial environments stems from the need for a computer vision technique that provides better and more accurate information about the target object to be inspected. In warehouses or manufacturing plants, the target objects to be inspected usually come in fixed positions, e.g., in roller belts with minimal degrees of freedom for variable positions, with controlled illumination sources, and in delimited spaces. The environment where objects are inspected is highly structured and well-equipped with sensors throughout the infrastructure.

The rise of inspection activities *outside* of these environments has led to the adoption of new vision and detection techniques that enable the understanding of the scenarios to be inspected in unstructured environments (but in a more sophisticated manner). In these unstructured scenarios, one cannot predict many of the objects that will appear in the scene during the inspection. This has led to the introduction of object detection using convolutional neural networks. AI-based object detection is useful for understanding what objects appear in an image, describing *what* is in it (with a better understanding of the scene, classifying the objects individually and discriminating one from another), and *where* those objects are located in the image.

## 3. Proposed AI-Assisted AMR and AGV for Inspection Tasks

**Figure 1:**
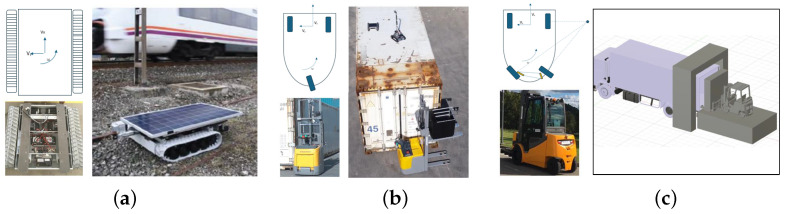
Robot motion types and use cases considered in our research: (**a**) caterpillar-based motion for rail inspection; (**b**) back-steering (wheel) for TEU container inspection; (**c**) back-steering, Ackermann, for the automated load of goods into trucks. Source: ZeniaLabs AI.

In our research, we focus on three use cases, each addressed by designing three different AMRs/AGVs (as shown in Figure 1a–c). One is a caterpillar track-based robot, and the others are two back-steering robots based on differently-sized robotized forklifts. The first one steers with one back wheel and the second one possesses a back Ackermann-wheel-based motion. This means that the steering is done from the back of the robot while the traction wheels on the front cannot steer but are in charge of moving forward and backward. After the full robotizing of the motion capabilities of the robot, they are provided with state-of-the-art kinematics, perception, robot localization, planning, and navigation capabilities. The most noticeable differences in the motion and navigational characteristics of the robots used in this research are that the rail robot can navigate on uneven, stoned terrain and can turn on its own axis. It is perfectly adapted for the rough terrain of the railway track’s environment where it has to navigate on uneven ballast and tracks.

These robotic platforms and the use cases themselves are described in Section 3.1, Section 3.2 and Section 3.3.

### 3.1. Use Case A: Rail Inspection and Maintenance

The first use case presented in this paper addresses the maintenance tasks for weed removal within the railway sector, which are crucial for the safe and proper operation of trains. Currently, most operators use the glyphosate herbicide for this task, spreading it around from maintenance wagons. This operation is performed at least twice a year throughout the entire worldwide railway network.

In order to find a solution that overrides the use of chemicals, a robotic AMR is designed. The combination of mobile robotics technology (in terms of mechatronics and conventional perception systems) with the introduction of AI object detection and classification algorithms, as a powerful and innovative high-precision sensor, offers an innovative combination for a new inspection task. It is considered the only alternative for detecting the number of infrastructure elements that must remain intact, as opposed to the growing vegetation and weeds between the railway rails that need to be detected, located, and eliminated.

**Figure 2:**
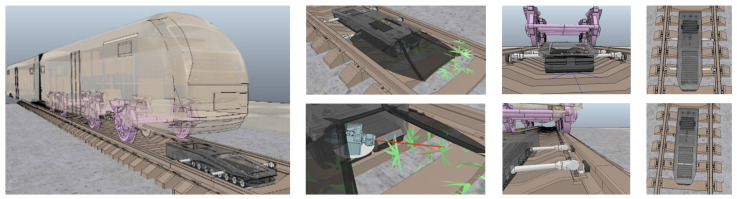
General concept design of the AMR designed to address Use Case A, including its flat design, to ensure co-existence with rail traffic; a general view of the robot’s restraint arms, enabling its attachment to the tracks when a train passes overhead; and weed detection and potential elimination from the robot. Source: ZeniaLabs AI.

Figure 2 illustrates the robot’s concept design, co-existing with rail traffic. The system is designed to accurately discriminate between weeds and, for example, cables, screws, or rail sleepers. Blue laser equipment is used for the elimination of the weeds once they have been precisely detected and located. In addition, the robot must operate without interfering with or stopping rail traffic. Therefore, it is flat enough to function under trains without colliding with the mechanical elements underneath. It also needs to withstand the suction forces from trains passing overhead. For this purpose, the robot was developed with lateral restraint arms that enable it to attach itself to the tracks when a train is passing.

### 3.2. Use Case B: Inspection of Maritime TEU Containers

The use case for maritime TEU container inspection was conceived to address safety issues in the inspection of TEU containers at ports. The inspection activities for these containers are crucial to ensure that once they leave the port to transport goods to customers, they are in optimal conditions for the cargo to be transported (watertight, clean, etc.). Moreover, it must be ensured that they are in perfect structural condition since any structural defects could lead to a fatal accident. For this purpose, inspections and, if required, repairs of the containers are performed worldwide each time a container reaches or leaves the port. In areas without an inspection gate, inspections are carried out by an operator who examines the asset and climbs to its top to visually detect and report any defects, holes, traces of corrosion, or any other structural damage he/she may find.

The inspection time directly affects the time the ship spends in port, so it should be kept as short as possible. This creates a situation where the inspection personnel suffer from timing pressure, leading to inefficiencies and inaccuracies in the inspection process, and eventually, human risks and potential injuries. The high-risk circumstances in which the inspection is performed are exacerbated when considering that such inspection work is done at height, with personnel climbing up ladders under strong winds (often in ports that are open to the sea).

The solution proposed in Use Case B consists of an automated inspection process supported by mobile robotics with integrated computer vision and AI algorithms that enable the smart inspection of maritime containers without the need to climb to the top of the container for a visual inspection.

**Figure 3:**
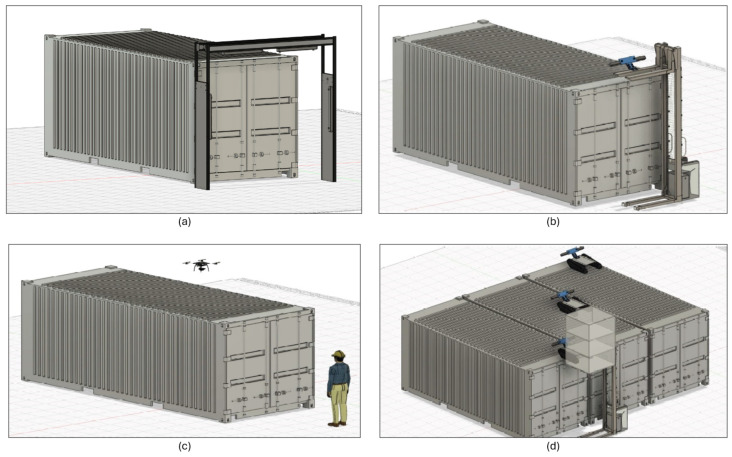
Automated inspection concepts for automatically detecting damages in TEU containers in maritime ports: (**a**) inspection gate; (**b**) forklift-based robotic solution; (**c**) sensorized inspection drone; and (**d**) proposed AMR approach. Source: ZeniaLabs AI.

The rationale for addressing Use Case B with AI-assisted robotics hinges on four potential scenarios, which are depicted in Figure 3:

- Scenario (a) in this figure is based on an inspection gate, which is the most simple and obvious way of inspecting defects without human intervention. It is a useful tool yet unfeasible for supporting inspection procedures outside specific inspection sites and areas where the gates are located. Usually, for on-site inspections, inspection personnel move to the port terminal, near the ships, to enhance agility and reduce time, thereby shortening the duration that containers remain in the terminal.
- Scenario (b) is instead based on a forklift-based robotic solution that allows the robot to move around the container while analyzing potential damages and the general structural health of the container being monitored. The main advantage of this solution is that the robot can move to the location of the container, e.g., near the ships.
- Scenario (c) is a sensorized flying drone that flies above the top of the containers, equipped with the necessary vision sensors and hardware to capture images of the monitored asset.
- Scenario (d) is the one focused on in this research; it comprises the fact that containers are sometimes placed next to each other, with little or no space between the container sides. The inspection should be performed in a very agile and safe manner. Using small inspection robots deployed by the robotized forklift system, several containers can be inspected concurrently, while the robotized forklift inspects the sides of the containers with its onboard cameras.

In all four scenarios, the methodology involves acquiring pictures and videos to be processed by high-speed electronics and generating a report in real time on the structural health of the inspected containers. The report needs to be available as quickly as possible on, for example, a handheld device. The main contribution of this research to this use case is focused on enhancing the robot’s perception with AI techniques for fully automated inspection of the containers.

Only scenario (d) has been developed, deployed, and tested for our research, justified by the fact that the containers to be inspected were located in an area where it was not feasible to install inspection arches or to operate drones. The drone scenario was discarded due to environmental restrictions at the test location (port of Bilbao, Bizkaia, Spain), which prohibited flying a drone without the appropriate permits from the airport authorities. The airport is too close to the test environment and poses a risk of interfering with air traffic. Despite these limitations, scenarios (b) and (d) are quite similar, with (d) being the evolution of the former and providing better insights on top of the container through the concurrent deployment of several small inspection robots. Moreover, we envision that scenarios (a) and (c) could be relevant for future research.

### 3.3. Use Case C: Automated Loading of Goods into Trucks

This third use case is related to the loading of goods into trucks, responding to the need to provide cargo workers with a system that allows them to delegate the final task of depositing the cargo onto the truck. Such a stacking process is not very ergonomic and is highly tedious, so our third use case involves *palletizing* the load outside the dock and then automatically transporting and depositing it inside the truck. At first glance, the challenge responds to a classic task where traditional computer vision could have been chosen. The infrastructure can be sensorized, and the robot can map it into a structured point cloud. Issues arise when entering the truck. Each truck is different inside, and may also differ from each other when parked at the loading dock. The main difference in the parking task is the angle and offset at which the truck driver parks the trailer. This is the point where Use Case C takes advantage of AI models to infer the exact position at which the truck has been positioned, the inner load status of the truck, and what space is available for proper deployment of the remaining goods. The interior space between the palletized goods and the truck walls may be less than 5 cm on each side, escalating the risk for the human operator and making the task of loading goods for an automated mobile robot even more challenging.

**Figure 4:**
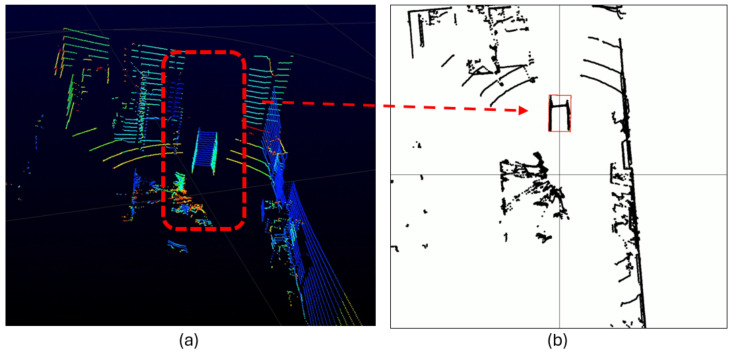
Examples of the detections from the LiDAR system used to address Use Case C: (**a**) LiDAR scans; (**b**) detection by the object detection model.

Unfortunately, no images from the warehouses’ field tests can be presented due to confidentiality clauses. Nevertheless, LiDAR images captured by our designed robot are depicted in Figure 4 to exemplify the information processed by the AI models in Use Case C. The overview of the LiDAR point cloud is processed by object detection models deployed on the robot to obtain the relevant details required for decision-making. To this end, the AGV robot is equipped with the appropriate perception systems to maneuver from the point of pallet collection to the trailer of the truck and back again. However, it also requires accurate information about the location of the loading dock, whether the appropriate trailer is there, and its pose (angle, lateral deviation). Once it enters the trailer, it maneuvers inside without colliding with the trailer sides.

## 4. Experimental Setup

**Figure 5:**
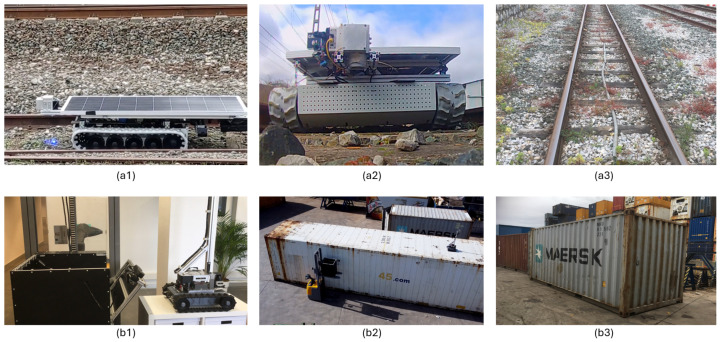
(**a1**,**a2**) Rail robot. (**a3**) Rail testing environment. (**b1**) Laboratory setup of the top inspection robot and side cameras. (**b2**) Field test setup in the port. (**b3**) TEU container for inspection activities.

Figure 5 presents an overview of the experimental setup for the rail and port applications. In Figure 5(a1), the rail robot in the operation of weed removal is presented. Figure 5(a2) presents a bottom view of the robot setup. In Figure 5(a3), the rail testing environment for AI benchmarking is displayed. In Figure 5(b1), the laboratory setup of the side cameras and small robot for inspection of the top of TEUs is depicted. Figure 5(b2) illustrates the field test setup in the port environment. Finally, Figure 5(b3) shows a TEU as the inspection environment for the AI benchmark presented in this study.

In order to analyze the benefits of using YOLO-based detection and classification algorithms for improving the robot’s performance, the necessary image acquisition hardware was mounted on prototype robots for the execution of experiments in laboratory and field test scenarios.

**Figure 6:**
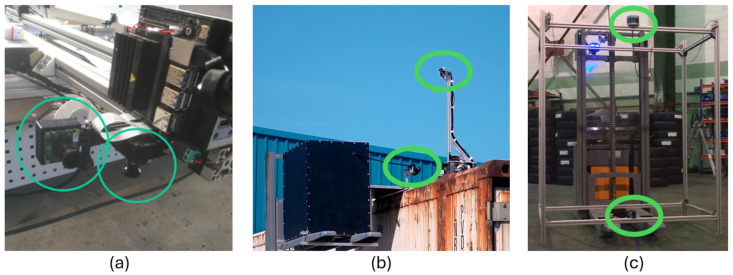
Overview of the sensors in the experimental setup of the three use cases, highlighting the cameras and LiDAR (laboratory environment) sensors for AI processing.

Figure 6 illustrates the main location of the image and lidar acquisition systems of the three proposed robotic solutions. The rail inspection robot holds two MIPI cameras (Figure 6a), one pointing at the front for obstacle detection and navigation, and another one pointing to the ballasted floor to detect and locate the weeds to be eliminated. The robot solution for TEU inspection is equipped with two stereo cameras: one on the robotized forklift to inspect the container sides and another on the small robotic rover deployed by the forklift on top of the container. The forklift moves around the container, and the rover navigates on the roof of the container, both acquiring images for AI processing (Figure 6b). Figure 6c presents the experimental prototype setup for the robotized forklift for loading goods into trucks. The location of a 3D LiDAR from RoboSense is highlighted, featuring 16 laser layers that generate a point cloud of the warehouse and inside the truck. The image processing first creates a zenithal bird-view of the scene to determine the pose of the truck in the docking bay. The AI processing system then decides whether the position is suitable for the maneuver of autonomously entering the trailer of the truck for unloading. The system calculates the angle and displacement of the trailer relative to the AGV coordinates to establish the correct entry and navigation trajectory and to plan the appropriate path. If at any point the pose is detected to be incorrect, the AGV initiates an emergency stop. Additionally, the AGV is equipped with a camera on the bottom to inspect the site for potential obstacles (such as fallen material or goods) in its path.

### 4.1. General Design for AI Integration

In our approach, the main goal in designing the object detector for the robots is to ensure it can respond within a reasonable time frame that matches the inspection needs. Therefore, we focus not only on the quantitative and qualitative capabilities of the model but also on the inference time to provide an answer. The model must successfully and accurately provide a positive response for detection within a limited time from the moment the robot navigates to or stops in front of the target.

In our design, we first analyzed the advantages and disadvantages of single-stage and two-stage detectors. On one hand, two-stage object detectors are very powerful and extremely accurate, achieving very high values of mAP. They are well-suited for domains where classification accuracy is more critical than speed. Examples of two-stage object detectors include the R-CNN family [25] or SPP-Net [26]. Two-stage detectors separate the tasks of object localization and classification for each bounding box. In contrast, one-stage detectors make predictions for object localization and classification simultaneously, which favors inference speed in real-time detections. Single-stage object detectors provide direct output of classification and bounding box coordinates from the input images. The images are processed through a feature extractor using a CNN, and the extracted features are then used directly for classification and regression of the bounding box coordinates. Therefore, single-stage object detectors are very fast and suitable for real-time object detection, though their performance may be compromised and can yield results that are inferior to those of their two-stage counterparts. The most representative one-stage object detection models are YOLO (you only look once) [27,28,29], SSD (single-shot multi-box detector) [30], and RetinaNet [31]. More recently, Transformers have also been used for object detection, with the so-called detection Transformer (DETR) architecture being the most renowned model relying on attention layers [32].

### 4.2. AI Modules from the YOLO Series under Consideration

Although the detection accuracy of one-stage detectors may lag behind that of two-stage detectors, they are better suited for the use cases under consideration, since they require real-time detection capabilities. The YOLO series has been the most popular detection framework in industrial applications, due to its excellent balance between speed and accuracy [33]. The intrinsic characteristics of the YOLO detector series, only requiring a single pass over each image, make them a suitable approach for the experiments.

We review the YOLO models’ architecture to better understand the advantages, disadvantages, and main differences between versions. We start with the YOLOv4 version of the YOLO family. It is not the first of the YOLO algorithms; however, it has been improved over its predecessors YOLO, YOLO9000, and YOLOv3, which laid the foundation for the ‘You Only Look Once’ detector family. The benefit of YOLOv4 is that its design focuses on prioritizing real-time detection. This is a key performance indicator for the robot and a critical factor for our research. We initiate a YOLO performance benchmark on object detection for the proposed use case with this module.

A crucial point in our detection requirements for real-time detection of the robot is conditioned by its AI processing capabilities. Without real-time processing power, the only chance to detect and classify any target objects is to record video frames for a subsequent offline analysis. However, this possibility is not considered because it clashes with the operational needs of the robot in the field tests and would only be useful in laboratory tests.

With the development of the YOLOv4 detector, Bochkovskiy et al. [34] aimed to improve the accuracy of real-time object detectors, enabling their use not only for hint-generating recommendation systems but also for stand-alone process management and reducing human input. Previous neural networks did not generally operate in real time and required a large number of GPUs for training, with a large mini-batch size. The architecture of YOLOv4 uses a CSPDarknet53 [35] backbone comprised of 53 convolutional layers. Within the neck part of the model, it uses PANet (Path Aggregation Network) [36] for feature aggregation of the network. Additionally, YOLOv4 adds a spatial pyramid pooling (SPP) block after CSPDarknet53 to increase the receptive field and separate the most significant features from the backbone. YOLOv4 employs a *Bag of Freebies* mostly focused on data augmentation, so termed because they improve the performance of the network without adding any inference time penalty in production. For data augmentation, the strategy uniquely incorporates self-adversarial training (SAT), aiming to find the portion of the image that the network relies on during training. Within the so-termed *Bag of Specials*, the authors used a type of non-maximum suppression (NMS) where Distance-IoU is used instead of regular IoU [37], cross mini-batch normalization (CmBN), and DropBlock regularization to significantly increase performance without adding a noticeable inference time penalty.

The next AI modules within our benchmark are YOLOv5 and YOLOv6. YOLOv5 was released only a month after its predecessor, YOLOv4 [38]. A significant modification that YOLOv5 included over YOLOv4 was the integration of an anchor box selection process into the model. Later, a target detection framework YOLOv6 was designed in [33] for industrial applications, featuring a strong focus on detection accuracy and reasoning efficiency. The resulting model, coined as Meituan-YOLOv6 (MT-YOLOv6), is not a part of the official YOLO series. Nevertheless, it has been referred to as YOLOv6 as it was inspired by one-stage YOLO algorithms. YOLOv6 improves over its predecessors by adopting an anchor-free paradigm that significantly enhances speed compared to anchor-based detectors. It also introduces a novel label assignment strategy to dynamically allocate positive samples, further improving detection accuracy, and includes an SIoU (SCYLLA-IoU) [39] bounding box regression loss to supervise the learning process of the network. SIoU is a variant of IoU that incorporates new cost functions. In terms of architecture, YOLOv6 utilizes an EfficientRep Backbone instead of the CSP-Backbone used by YOLOv5. YOLOv6 also replaces the common convolution layer with the RepConv layer and CSPBlock with RepBlock. These changes allow the object detection model to efficiently utilize the computing power of GPU hardware while maintaining strong characterization ability. Small enhancements in its architecture lead to considerable improvements in small target detection [40]. Regarding its internal architecture, the neck design of the YOLOv6 model features a Rep-PAN Neck to make hardware utilization more efficient and to achieve a better balance between accuracy and speed. It also offers a more effective feature fusion network based on a hardware-suited neural network design, along with a decoupled head that considers the balance between the representation ability of the relevant operators and the computing overhead on the hardware.

The next model in the performance benchmark across Use Cases A, B, and C is YOLOv7 [41], which is notable for its efficient E-ELAN layer aggregation. This aggregation is an extended version of the ELAN computational block designed to control the shortest longest gradient path. It relates to the amount of memory required to keep layers in memory and the distance it takes for a gradient to back-propagate through the layers. Furthermore, the network depth and width are scaled up while concatenating layers together, optimizing the model architecture for different sizes. YOLOv7 also utilizes gradient flow propagation paths to determine which modules in the network should use re-parametrization strategies.

Different from the previous YOLO generations, YOLOv8 [42] uses mosaic data augmentation that mixes four images to provide the model with better contextual information. The change in YOLOv8 compared to previous versions is that the augmentation stops in the last ten training epochs to enhance performance. Also, the model switches to anchor-free detection to improve generalization, directly predicting an object’s midpoint and reducing the number of bounding box predictions. This speeds up NMS to discard incorrect predictions. To accelerate the training process and enhance gradient flow, the model’s backbone includes a C2f module, where the model concatenates the output of all bottleneck modules, unlike the C3 module in previous YOLO models, which utilized the output of the last bottleneck module. A decoupled head performs classification and regression separately.

Finally, the benchmark is completed with YOLOv9, the latest in the series of YOLO object detectors to date. In their recent presentation to the community [43], Wang et al. proposed a new concept called *programmable gradient information* (PGI). PGI addresses the information bottleneck problem without data loss in the feed-forward process. The model generates reliable gradients via an auxiliary reversible branch. Deep features still execute the target task and the auxiliary branch avoids the semantic loss due to multi-path features. The reversible architecture of PGI is built on the auxiliary branch, so there is no additional computational burden. YOLOv9 has shown strong competitiveness, reducing the number of parameters and the number of calculations when compared to the state-of-the-art.

Table 1 depicts the number of layers and internal parameters for the different versions of the YOLO models considered in our benchmark.

### 4.3. Evaluation Protocol and Hyperparameters

All YOLO models described in the previous subsection have been used to address the use cases under consideration. This benchmark allows for comparing their performance in detecting objects across different inspection scenarios. We will now describe the datasets collected in every use case for training and evaluating the performance of the object detectors:

Use Case A

The use case for rail maintenance and weed removal is trained on three classes for detection: (1) the weeds that need to be detected, classified, and located for elimination within the rail tracks; (2) the cables within the rail tracks, which are part of the rail infrastructure and must not be damaged under any circumstances; and (3) other objects to be found on the tracks, like bottles, plastic bags, or cardboard pieces. Such objects (and others alike) can be essentially ignored for inspection purposes. However, it is positive to detect them and inform them about their presence and location to the railway operators. This is especially true when dealing with paper and cardboard, which can be dangerous when weeds are removed via laser if the beam hits them too long, creating the risk of starting a fire.

The training dataset collected for this first use case is composed of 3736 images containing objects of the three classes (9216 weed objects, 5742 cable objects, and 733 other objects). The most prevalent objects are weeds, followed by cables and certain sporadic appearances of foreign objects. The dataset has been preprocessed and augmented via different transformations to increase its diversity and potentially yield a more generalizable object detector. We also created a version of the dataset with only weed annotations to evaluate whether the object detector, when focused solely on the most relevant label for the inspection task, achieves better performance figures. The preprocessing transformations of the images include auto-orienting and stretching each image to 640×640 pixels, a static crop between 25% and 75% in both horizontal and vertical ranges, a gray-scaling operation to make the models faster and insensitive to subject color, adaptive equalization to improve normalization and line detection in varying lighting conditions, and splitting the images into tiles to enhance accuracy on small objects, which is beneficial for cable detection due to the small and confined space of the cables within the rail.

The data augmentation strategy in this first use case includes flipping the images horizontally and vertically, as well as ±15 degree random rotations to make the model more insensitive to object orientation. Added image modifications include 2.5-px random Gaussian blurring to enhance resilience to camera focus issues, saturation adjustments (±25%), over- and under-exposure (±10%), and brightness adjustments (increasing the picture or darkening it by 15%). Moreover, noising the new images by 0.1% aims to make the model more resilient to camera artifacts. These steps have resulted in a dataset totaling 35,896 images. It has been split into 88% (31,428 images) for the training dataset, 8% (2960 images) for the validation dataset, and 4% (1508 images) for the test dataset.

Use Case B

A similar preprocessing and data augmentation procedure has been performed for Use Cases A and C. Use Case B comprises a dataset of 1035 images, split into a train–val–test proportion of 70%–20%–10% (992 images for training, 35 images for validation, and 18 for test purposes). The defects that the AI models discover are bowed panels, holes, and oxide. The defects may come together in the same scene in some cases. As a result, three different classes are annotated in the images: oxide (232 objects), hole (73 objects), and bow (41 objects). Preprocessing and augmentation are applied to the training set to minimize the spurious effects of light, contrast, visual noise, and other camera-related effects. After augmentation, the training set of images increases to 6200 images.

Use Case C

This third use case handles the images differently. The input data come from LiDAR point clouds and are converted into grayscale images for analysis. The goal is to find the correct or anomalous pose of the truck in the bay according to the synthetic images received from the LiDAR sensor. Therefore, the two classes trained within the model are (a) correct, standing for the correct pose of the truck, and (b) anomalous, denoting incorrect poses, where the AGV triggers an emergency stop. After converting all point clouds to images, the dataset consists of 10,667 images with 9082 correct pose objects and 534 anomalous pose objects. This dataset is split into a 70%–20%–10% stratified proportion, yielding 6754 images for training, 1919 images for validation, and 943 for testing. Neither preprocessing nor augmentation is required for this dataset since they are LiDAR point cloud grayscale images with few camera-dependent features. Consequently, the original dataset suffices for training an object detection model with good generalization capabilities, as later shown by our reported results.

Training Hyperparameters

Models were trained on a Colab Jupyter Notebook using an NVIDIA T4 GPU. The training parameters were set to T=100 epochs and a batch size of B=16. Values of the rest of the hyperparameters are listed in Table 2.

For the most recent YOLO models (from V5 onward), internal data augmentation is performed. For our experiments, such augmentation includes HSV (hue, saturation, value) with fractional parameters hsv-h=0.015, hsv-s=0.7 and hsv-v=0.4; image translation with fractional parameter translate=0.1; and scaling with parameter scale=0.9. Neither shearing nor perspective-changing was set. Flipping left–right (horizontally) augmentation was configured with parameter fliplr=0.5, image mosaic generation had a probability of 1.0, image mixup augmentation had a probability of 0.15, and CutMix augmentation was configured with parameter copy-paste=0.3.

Evaluation Metrics

We consider standardized metrics for this particular modeling task [44], specifically the Precision and Recall values, the mean average precision at a specific Intersection over Union (IoU) threshold of 0.5 (denoted as *mAP@.5*), and averaged over IoU thresholds from 0.5 to 0.95 in steps of 0.05 (*mAP@.5:.95*), and the *F1 score*. The Precision score informs about the quality of a positive prediction made by the model by counting the number of true positives divided by the total number of positive predictions. The Recall metric denotes the fraction of correctly classified positive cases. With the Recall metric, we capture the positive cases that are misclassified by the model as negatives. It is an important metric in our use cases for the criticality of certain cases in each application, where a false negative can have damaging consequences. This is especially true for Use Case A if the robot incorrectly shoots at cardboard with the weed-removing laser, and for Use Case C, where an incorrect detection of the truck’s pose could result in a collision with the AGV. The mean average precision metric *mAP* measures the average precision of detection across all classes in the model. It is evaluated at two different detection thresholds. Firstly, it measures precision at a threshold where the positive detection aligns with the ground truth within the selected intersection range, called the *intersection over union (IoU)* of 50% (mAP@IoU = mAP@.5). The second metric, mAP@.5:.95, is much more restrictive and gives the values of the positive detections within an intersection range between 50% and 95% of the detected object according to the ground truth. Finally, the *F1 score* measures the performance of the model by combining its precision and recall scores as 2·Precision·Recall/(Precision+Recall).

## 5. Results and Discussion

Using the experimental setup and evaluation protocol described previously, we now present and discuss the results obtained for each of the use cases, namely, A (rail inspection, Section 5.1), B (TEU container inspection, Section 5.2), and C (truck pose estimation for automated loading, Section 5.3). We end the discussion with a qualitative analysis of the produced detections for all use cases in Section 5.4.

### 5.1. Results for Use Case A

The results obtained for this first use case are summarized in Table 3 (one object class) and Table 4 and Table 5 (three object classes). We report the performance metrics scored by the YOLOv4 to YOLOv9 models over the test dataset collected for the use case.

We begin by discussing the results of the binary detection model. The model was trained to only find weeds on the rail tracks, without detecting any other object class. The model specializes in weed detection, yielding very good detection metrics for all YOLO versions considered in the benchmark. The field tests demonstrate high performance, as evidenced by the metrics in Table 3, reporting values as high as 0.96 for mAP@.5 and an F1 score of 0.972 for YOLOv9. Due to the specialization of the trained model to the weed class, its application is strictly limited to the Use Case and its environment. Consequently, it has no further utility outside the scope of the rail environment.

We follow by considering the alternative experiment in which the model is trained to detect weeds, cables, and other objects (e.g., bags, cardboard) within the rail tracks. We first focus on evaluating the performance of the model across all three classes and compare it to the performance of the model specialized in weed detection. As shown in Table 4, detecting and classifying more than one class with a single object detector involves a substantial overall decrease in the performance metrics for all models. We further inspect the detection results by analyzing what affects the overall performance and how such performance degradation spreads over the object classes considered in this second experiment. This is the purpose of Table 5, which breaks down the performance figures for the three classes and the best-performing YOLOv9 model. The main objective of the model is to detect weeds. The relatively higher presence of annotated weed objects in the collected dataset makes this class well-represented. However, the high visual variability of weeds and the model having to detect the other two classes give rise to lower detection scores for this class. Nevertheless, precision and recall are well balanced, as opposed to the scores associated with the other class. In this second class, the recall degrades significantly, meaning that many objects belonging to the other class are not detected by the model. The third cable class, however, undergoes severe degradation of both precision and recall. Cables laid all over the train route are well-detected by the model. However, the model not only fails to detect individual cables (low recall) but also produces false positives (low precision) due to the inherent difficulty of detecting filament shapes in real-world image data.

### 5.2. Results for Use Case B

Results of the performance evaluation of the YOLO models for detecting defects in TEU containers are summarized in Table 6. Despite the a priori difficulty of identifying such defects in practical settings, the model scores particularly high compared to the other two use cases, considering that it accounts for three defect classes. Intuitively, the variability and number of possible defects on a large flat panel surface with shadows and sometimes poor illumination pose a significant challenge for AI-based object detectors. The reason for the positive results in our setup lies in the high contrast of the specific defect classes targeted in TEU containers against other parts of the images. For example, oxide spots located on the container surface are well-matched by the model, as they are clearly distinguishable given the container’s color. Holes in the containers can also be well-detected thanks to their contrasting black color and mostly round shape, which stands out against the metal panel background. Containers are mostly white, blue, or red, and no tests on black containers were performed. Oxide defects are better detected on white and blue containers for obvious reasons.

### 5.3. Results for Use Case C

In the use case for warehouse pose detection (Use Case C), we first acquire a full view of the LiDAR beams captured by the robot (exemplified in Figure 4). There, we can recognize the shapes and structures of the docking bay where the robot has to navigate to. It is of high importance that the robot correctly points to the truck in the bay with the correct pose, so it can autonomously enter with the load. Therefore, our pipeline trims the relevant segments from the whole LiDAR image to extract and process the pose of our robot. Through AI processing, the system decides whether the pose is correct or if the robot is in an anomalous position, yielding the detection scores shown in Table 7.

Due to the simplicity of the grayscale images resulting from LiDAR preprocessing, training YOLO algorithms on these images yields exceptionally good object detection models, achieving performances of mAP up to 84% at an IoU of 50%:95% (with YOLOv9). This is the highest score obtained in our experimentation and exposes the practical relevance of signal preprocessing in achieving actionable levels of performance without requiring convoluted modeling choices.

### 5.4. Qualitative Analysis

We finish our discussion of the results by examining some examples of the images captured in each of the use cases under consideration. To this end, we will complement this qualitative analysis with the F1 confidence curves associated with each of the classes annotated in each use case. This curve is a graphical representation used in binary classification to evaluate the performance of a model across different confidence thresholds, which determines the cutoff point for classifying instances as positive or negative. The confidence (x-axis) is computed as a combination of the conditional probability of the class given that an object has been detected, and the probability of a bounding box containing an object. This measure of confidence balances between how certain the model is that a box contains an object and how certain it is about which class the object belongs to. The y-axis represents the F1 score achieved by the model at each confidence threshold. By varying the confidence threshold, the F1 confidence curve illustrates how the trade-off between precision and recall changes. A higher threshold typically results in higher precision but lower recall, while a lower threshold leads to higher recall but lower precision.

**Figure 7:**
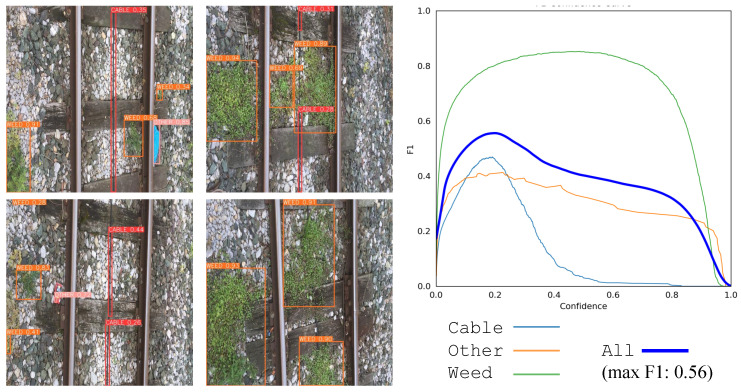
(**left**) Examples of detected objects by YOLOv9 over Use Case A, and (**right**) F1 confidence curve for weeds, cables, and other objects.

We begin the qualitative inspection of the results with Use Case A, in which models have been shown to encounter difficulties in detecting certain objects (classes). Figure 7 depicts snapshots of the detected objects by YOLOv9 (left), and the F1 confidence curve for each label (right). Cables within the rails are more likely to be detected with less confidence by the weed detector model at higher intersection-over-union (IoU) thresholds. The reason lies in their shape. Higher IoU values impose more restrictive requirements for location, and the long, narrow, and sometimes curved shape of the cables within the tracks poses a significant challenge for AI models to accurately locate the correct position of the boxes according to the ground truth. However, it must be stated that the cables can be correctly detected as true positives when they appear in the images, with an acceptable mean average precision at lower IoU levels (mAP@.5). This detection threshold is good enough to know that there is a cable in the scene and the model offers approximately good precision in its position so that the robot can avoid damaging the area close to it.

**Figure 8:**
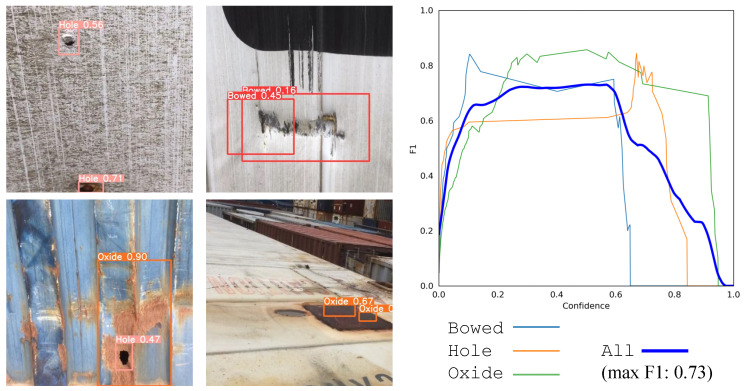
(**left**) Examples of defects detected by YOLOv9 over Use Case B, including holes, bowed panels, and oxide on top of the monitored asset; (**right**) F1 confidence curve of each class.

**Figure 9:**
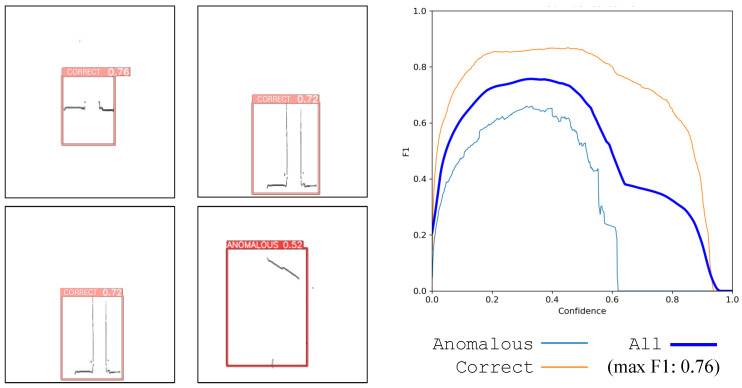
(**left**) Exemplifying instances of Use Case C where YOLOv9 detects correct or anomalous truck poses; (**right**) F1 confidence curve for each class.

We proceed now with the results corresponding to Use Case B and Use Case C. In the four pictures on the left of Figure 8, we depict the detection of YOLOv9 for different types of defects, including bumped and holed containers with oxide parts on them. The right plot depicts the F1 confidence curve corresponding to each class, together with the one corresponding to all objects. It can be observed that all objects seem to be well-detected by the YOLOv9 model since the metallic surface of the TEU containers is chromatically less heterogeneous than the scene in Use Case A. Finally, the qualitative results of Use Case C are illustrated through examples with several correct and anomalous detected poses in Figure 9 (left), together with the F1 confidence curve of both classes (right). In this case, the examples on the right highlight the practical utility of preprocessing the LiDAR point cloud data (as shown in Figure 4) to remove contextual variability, allowing the model to receive only information that is predictive of its commanded task (the borders of the truck).

### 5.5. AMR Deployment, Achieved Operational Gains, and Practical Limitations

The preceding subsections analyzed the performance of the object detection models deployed on the AMR solutions developed for the three use cases under study. However, in practical real-world scenarios, the utility of an object detection model deployed on an AMR is not solely restricted to achieving actionable levels of predictive accuracy in benchmarks. By quantitatively measuring metrics such as processing speed and resource consumption, stakeholders can gain a comprehensive understanding of the model’s effectiveness, robustness in diverse conditions, and ease of deployment. Such an analysis is essential for identifying potential improvements, ensuring the safety of the design, and optimizing the robot’s performance in the targeted inspection tasks. To this end, this subsection describes the results of field test trials conducted with robots equipped with vision sensors and object detector models in real-world settings, under the framework of the European ESMERA project funded by the European Commission (ref. 780265), stressing key practical aspects that were validated on-site during the trials.

Use Case A

We begin with Use Case A, where we recall that the main objective of the AMR solution is to avoid the indiscriminate use of glyphosate. Currently, glyphosate is used at least twice a year, sprayed from maintenance trains traveling at 50 km/h. Such maintenance duties negatively impact the regular schedule of railway traffic. The novelty resides in using a non-chemical, non-polluting method (especially since there are efforts to ban glyphosate by regulatory institutions), which could be mechanical or, as proposed in this work, by laser irradiation. Undoubtedly, the robotic method is slower than the current one, but it aligns better with the search for clean methods.

**Figure 10:**
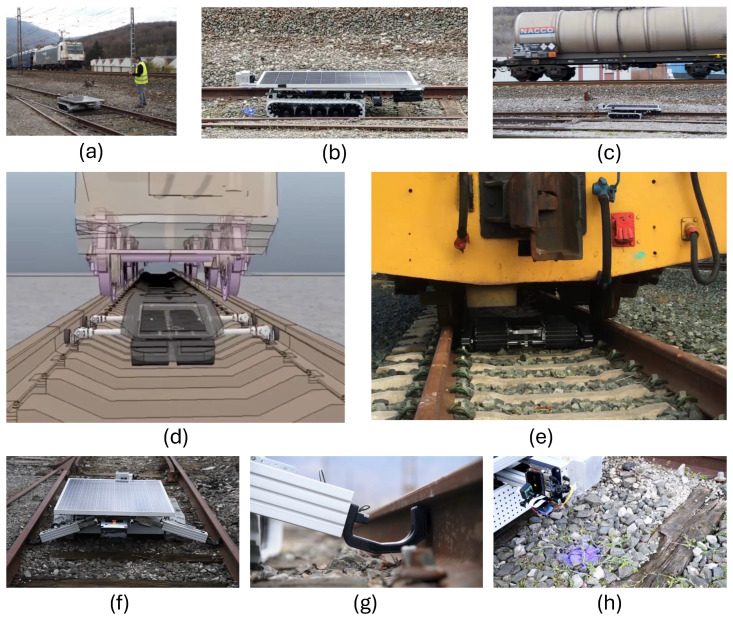
Images summarizing the field trials completed for Use Case A: (**a**–**g**) AMR solution set on the railway, showing that it mechanically adapts to the width of the railway; (**h**) detection and weed removal performed by the robot’s head.

Once the robot was deployed and run on the field tests designed in the aforementioned project (refer to the subplots in Figure 10 for a visual summary of the process), several key performance indicators were registered. From the mechanical point of view, the deployed AMR achieved a speed of 5 km/h, with an average power consumption of less than 2 kW (including laser, sensing, navigation, and processing systems). From the observed maneuvers, an average estimation of 3–5 s per plant was needed for eliminating a single detected weed, yielding a daily weed removal rate of the robot in the range of 17,000 and 120,960 plants/day. This estimation was made by taking into account the area radiated by one laser head, and the possibility of implementing an array of laser diodes or laser heads, with seven heads operating simultaneously on the rail track. These statistics depend on the railway’s status and the spatial distribution of weeds along the rail. Nevertheless, they serve as a good estimation of the practical benefits when compared to manual removal.

Further gains were observed during the trials. The laser procedure prevents weeds from growing up again at least in the next 4 months after the intervention. From the mechanical side, the AMR system safely engages to the tracks and delivers feedback in less than 1 min, ensuring its fast deployment. It also surpasses infrastructure items on the tracks lower than 30 cm in height. Infrastructure items on tracks that the train can pass over can be surpassed by the robot in its normal operation. Finally, the AMR carries a variable number of batteries (i.e., in attachable wagons to increase the navigational autonomy), so that it can work during a complete working shift (8 h) without recharging or changing the battery packs.

Apart from radically changing the weed removal method (from manual to automated), the use of YOLO algorithms was proven to be differential in detecting vegetation precisely. With conventional vision algorithms (SIFT/SURF/Harris/Hough for reference point extraction, and chromatic masking to discriminate among colors, all implemented by using the OpenCV software library), the false positive rate was at least 20% higher, posing a high risk of irradiating glass, cardboard, or plastic with the laser. The OpenCV algorithm overly relied on the plant’s chromatic (green) component and was excessively permissive to the morphology of the vegetation (overgeneralized). In other words, it did not effectively suppress false positives. YOLO handles much better cases in doubt, reducing the number of false positives in their detected objects by at least 20%.

Use Case B

**Figure 11:**
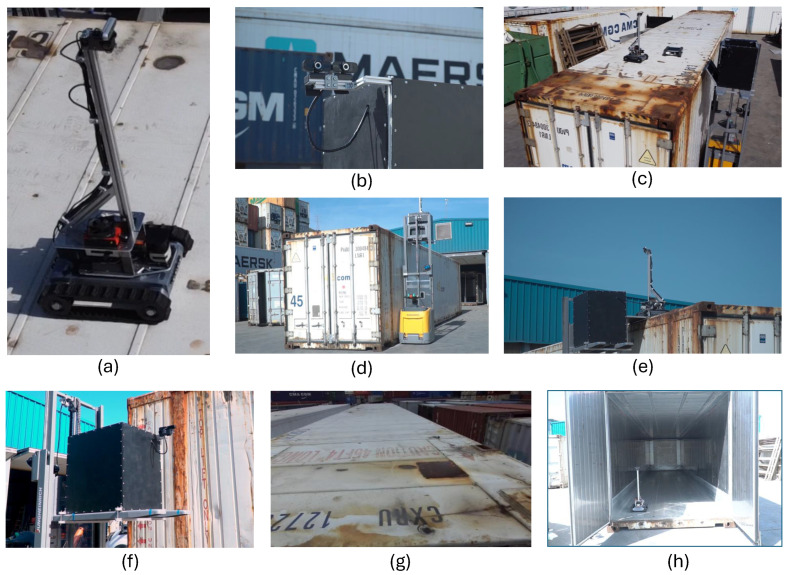
Summary of the field tests conducted for Use Case B: (**a**) implemented AMR device; (**b**) vision sensor on board; (**c**–**g**) different pictures of the robotic attachment process; (**h**) image showing that the devised solution can be used to inspect containers from the inside.

The usual procedure for the inspection of the containers first requires that the AGV navigates to the container. Once there, the AGV circles around the container (Figure 11c) in the first inspection, with the cameras installed in the liftable receptacle (black box) (Figure 11b,f). At a specific point, the elevator stops, opens the deployment ramp, and lets the surface inspection robots exit to the top of the container (Figure 11c). It first releases a robot from the first floor of the receptacle, then raises the elevator again to let the second robot go out. As it elevates, the side cameras of the receptacle acquire lateral images (Figure 11e). The robots concurrently inspect the top of the container (Figure 11a,c,g), while the AGV continues to circle the container, concurrently inspecting the sides, while the robots on top inspect the surface of the container. Finally, the AGV raises the lift pod again to pick up the robots. It opens the access ramp, and the first robot enters the receptacle. It then lowers the receptacle slightly, deploys the second ramp, and the second robot enters it (Figure 11e).

In the second use case, the field trials showed unexpected limitations of the devised solution: the AMR was unable to inspect the sides of containers that were adjacent to each other, even with conventional (visual) manual inspection. In this case, the following steps were taken:

- The container targeted for inspection was separated from the others to allow access to its sides. In the port terminal, containers were in constant motion as they were loaded and unloaded from ships. Therefore, while this container movement slowed down the inspection and was more inconvenient, it was not a critical maneuver for the port operations.
- If the container was empty, it was inspected from the inside for light leaks (Figure 11h), indicating the presence of a hole. This workaround only allowed identifying hole defects.

As a result of our field trials in Use Case B, defects could not be detected by the AMR more effectively than by the port experts. The port premises house very experienced operators who directly understand the potential causes of each defect. However, the method did achieve one of the desired safety outcomes by preventing them from climbing to the top of the containers, which was one of the desired outcomes in terms of safety. Also, by automating the process, we enhanced the digitization of the entire process and the data, because images sent and stored by the system are useful for the traceability of the inspection process and the accountability of decisions made. In all cases, operators decide whether to remove a container from circulation and set it for repair. However, the developed AMR system provides an informational database that can be used to safely validate such decisions.

From a mechanical perspective, one of the biggest limitations identified during the trials emerged when the upper robots moved from one container to another from the top. The initial idea was to let them move on their own in areas with many containers placed close to one another, traversing across all the containers by navigating through the small gaps and spaces between them. This did not work as expected. Although containers were close enough together (causing the infeasibility of a lateral inspection, as noted above), there was too much space for the top AMR to move from one container to the next one by solely relying on their tracks without falling or becoming stuck between the two containers. To amend this issue, the containers should have been placed less than three or four centimeters apart, but many of them were slightly more separated than this critical distance. The underlying trade-off between the maneuverability of container deployment in the port premises and the autonomy of the AMR to navigate through contiguous assets has captured the interest of the management of the port authority and is expected to drive applied research studies in the future.

When it comes to the object detection model itself, a common problem occurred with containers damaged with large dents, i.e., those covering almost the entire side panel of a container. Models trained to identify those defects effectively ended up detecting any container structure as a defect, annotating any panel as such. The reason for this detection failure is twofold: (1) the visual information varies significantly when the dent is viewed from different angles, which can be challenging even for the human eye; and (2) there is not as much chromatic information as when the dent is small, e.g., a scratch removing a significant amount of paint from the container’s surface. We envision that for this particular type of defect, the AMR should be equipped with additional sensors, increasing the cost of the overall robotic approach.

Despite these unexpected eventualities in the test trials of Use Case B, they demonstrated the improved safety of automating the entire operation rather than doing it manually. The key for port operators to embrace this solution was the incorporation of AI-empowered object detection models for the defects; otherwise, the performance differences compared to visual inspection would have been too significant for the AMR-based approach to be of any practical usefulness.

Use Case C

The cargo transport operation tackled in Use Case C involved a maneuver that none of the operators wanted to perform. They had to drive the AGV so close to the walls of the truck (where they could hardly see anything) that very few of them had managed to do it without bumping into the sides of the cargo truck. Most operators typically struggle with orientation; they start moving the forks inside, but often end up getting stuck inside the truck, requiring many maneuvers to deposit the load. Only minimal correction maneuvers are possible inside the truck, both laterally and angularly. The angle must be precisely defined before entering, taking into account that the truck is not always positioned the same way in the bay: there is always some lateral and angular displacement that complicates the loading maneuver. The trucker parks it with some references to the bay, but there is always some displacement. For manual loading, this displacement is irrelevant. However, for the AGV to operate autonomously, it is crucial that the maneuver is planned in advance. In this case, the AI-based object detector indicates whether the AGV is correctly aligned with the trailer. Upon a positive response, we can then calculate the angle at which the truck has been docked, in order to adjust the AGV’s pose to match the truck’s angle. The object detector aids in identifying the shapes within the point cloud that are characteristic of the bay entrance and the rear of the trailer, as well as indicating whether it is correctly oriented.

**Figure 12:**
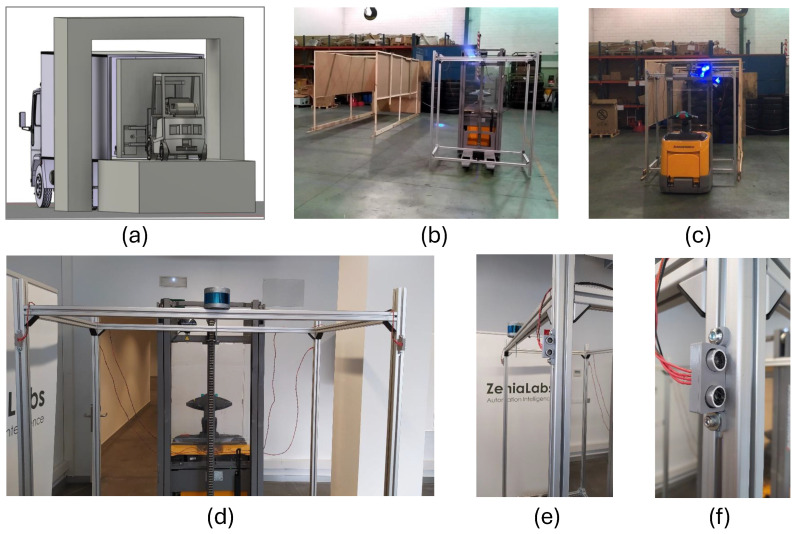
Images illustrating the field tests performed for Use Case C, from (**a**) the concept to (**b**–**f**) the experimental setup constructed for validation.

To verify the operational performance of the robotic solution devised to address this use case, a metallic structure was constructed to simulate the load to be deployed by the AGV inside the trailer (Figure 12a–d). Once inside the trailer, measurements were taken with the lateral ultrasound and LiDAR sensors installed in the structure (Figure 12e,f). It should be noted that in a fully real-world scenario, the same sensors are located in similar positions on a palletizer rather than on the aluminum frame used in our experiments. In addition, the robotic forklift is a pallet truck with a higher load capacity (Figure 1c) because it must lift several tons.

In this case, once the robot has entered the truck perfectly aligned and with the correct orientation, it adjusts 1 cm at a time inside the container, moving slowly but steadily until it deposits the load. When exiting, it proceeds likewise in reverse. This is only possible if perfect orientation and movement are ensured upon entering. In our field trials, the correct or anomalous pose of the truck in the bay, detected by the AI-based approach from the images generated from the point cloud data captured by the LiDAR sensor, was found to be very valuable in safely completing the loading maneuver. However, in the field tests, the machine also failed to autonomously correct minimal angular and lateral deviations inside the truck. Despite the slow-motion dynamics imposed on the robotic solution (1 cm per cycle), the correction was not successfully completed in several spatial configurations. As a result, the AGV ended up hitting the lateral panels of the truck, causing catastrophic structural damages due to the high inertial force of its load. In such cases, the solution was given by the detection of a collision by proximity based on the ultrasound sensors, triggering a stop emergency in the AGV. The pose estimation method (based on YOLOv8, which elicited the best detection performance for this use case) and the correction prior to the entrance maneuver were found to be the only effective ways to perform a correct entry maneuver of the AGV into the truck. This, combined with the lateral collision avoidance system, comprised the overall AGV automated maneuver system that led to satisfactory results in the conducted field tests.

## 6. Conclusions and Future Research

In this work, we evaluated the potential of AI-based object detectors for supporting inspection tasks performed by autonomous mobile robots and automated guided vehicles. Specifically, we assessed the detection and classification performance of the YOLO series for object detection in image data collected by AMR and AGV across three different real-world setups. Inspection tasks performed by robots in such scenarios can only be achieved through the introduction of an AI algorithm capable of selecting, detecting, and classifying targets in the scene that are critical for the inspection to be conducted by the robots. We have demonstrated that the YOLO detection family is well-suited for such tasks, given that the models have been specifically created and trained to detect and identify objects from images. Our discussion on the performance of such object detectors has been complemented with a description of the field tests for the three targeted use cases, verifying the practical usefulness of AMR devices equipped with such AI-based functionalities, and unveiling unexpected limitations and challenges to be addressed in follow-up studies.

We note that the good performance of the models is the result of them being trained for detection that is specific to the purpose and environmental context of the inspection tasks. Models produced in our research validate that AI models for object detection can be excellent tools for robotic perception. However, their generalization to other contextual conditions beyond those in the use cases considered in this study may result in a degradation of performance. Furthermore, when considering more classes to be detected by the learned models, we have found that the general performance, measured in terms of mean average precision for a specific IoU threshold, degrades considerably.

Our main conclusions drawn from our results can be summarized as follows:

- In Use Case A (railway inspection), the goal of the AMR is to detect weeds between the railway tracks so that they can be eliminated by a laser beam. This mechanical solution can be an alternative to the current method using glyphosate, which is spread from maintenance wagons or trucks. An AMR supported by AI-based detection has been shown to perform effectively in discriminating between weeds to be eliminated and other critical rail infrastructure objects (e.g., beacons, cables, and debris), which the robot must detect to avoid causing damage.
- In Use Case B, the complexity of detecting and classifying specific defects in TEU containers is currently achievable only by human workers. The challenges and dangerous situations associated with such tasks are considerably well supported by AGVs and camera-based AI object detection, where robots equipped with cameras are deployed at the inspection site. The detection results in this second use case have also been promising, mainly due to the better discriminability of defects on the metallic panels of the TEU containers under inspection.
- Finally, when it comes to Use Case C (automated loading of goods in trucks), we have proven that this risky task can be fully automated by a robotized AGV. In this case, the support of AI models is required in addition to the robot’s perception system. This support ensures that a docking maneuver is initiated from the correct position and that the AGV does not start from an anomalous position, which could potentially lead to an incorrect trajectory. The inclusion of AI in this task provides enhanced capabilities beyond what traditional computer vision systems can achieve.

On an overarching note, our threefold benchmark of YOLO object detectors over real-world data and the results of the field tests with robotic platforms equipping them have proven the feasibility of these AI-based models as an additional, high-accuracy perception sensor for robotic inspection tasks.

Several research directions can be pursued in the future based on the findings made in this work, beyond overcoming the practical limitations noticed during the field tests. Among them, we highlight the need to ensure proper generalization of the performance of the learned model across similar inspection scenarios that are subject to different environmental artifacts at their input (e.g., different characteristics of the asset) and/or their output. Regarding the latter, an interesting line to follow is to explore the possibilities of class-incremental learning [45] to make the model autonomously detect and characterize new objects in the scene. To this end, we plan to investigate whether uncertainty estimation techniques for object detection [46] can be exploited and used to detect unknown objects that appear recurrently over time, so that the model can consolidate them as a new class to be discriminated.

## Figures and Tables

**Table 1:** Variants of each YOLO model considered in the benchmark, alongside their trainable parameters, layers, and floating-point operations (FLOPS).

Model	Variant	# Layers	# Parameters	FLOPS
YOLOv4	YOLOv4-l	334	64,363,101	142.8 G
YOLOv5	YOLOv5-m	290	21,172,173	49 G
YOLOv6	YOLOv6-m	156	34,900,000	85.8 G
YOLOv7	YOLOv7-l	314	36,492,560	104.7 G
YOLOv8	YOLOv8-m	365	43,632,153	165.2 G
YOLOv9	YOLOv9-c	621	25,439,385	102.8 G

**Table 2:** Hyperparameter values used in the experiments.

Notation	Value	Definition
lr	0.01	Learning rate of the stochastic gradient training solver
β	0.94	Momentum for the stochastic gradient training solver
η	5 × 10^−4^	Weight decay parameter ($L_{2}$ penalty)
$\left(\beta_{0},lr_{0},T_{0}\right)$	(0.8,0.1,3)	Initial β and lr values that ramp to the default values over $T_{0}$ epochs
$\left(G_{□},G_{cls},G_{BCE},G_{DFL}\right)$	(7.5, 0.5, 1.0, 1.5)	Box, classification, BCL, and DFL loss gain (YOLOV8)
$W_{BCE}^{+}$	1.0	BCE loss positive weight
$IoU_{\min}$	0.2	IoU training threshold
$\gamma_{am}$	5	Anchor-multiple threshold (YOLOV5)

**Table 3:** YOLO performance comparison in Use Case A when detecting object instances of only one class (weed). The best score values are shaded in gray.

YOLO Version	Precision	Recall	mAP@.5	mAP@.5:.95	F1 Score
YOLOv4	0.912	0.978	0.974	0.491	0.944
YOLOv5	0.929	0.957	0.956	0.513	0.943
YOLOv6	0.964	0.634	0.964	0.529	0.765
YOLOv7	0.823	0.753	0.787	0.320	0.786
YOLOv8	0.968	0.983	0.976	0.612	0.975
YOLOv9	0.966	0.978	0.966	0.594	0.972

**Table 4:** YOLO performance comparison in Use Case A when detecting object instances of 3 classes (weed, cable and other).

YOLO Version	Precision	Recall	mAP@.5	mAP@.5:.95	F1 Score
YOLOv4	0.515	0.562	0.483	0.303	0.537
YOLOv5	0.663	0.546	0.540	0.334	0.599
YOLOv6	0.528	0.472	0.405	0.279	0.498
YOLOv7	0.658	0.531	0.491	0.344	0.588
YOLOv8	0.709	0.556	0.590	0.420	0.623
YOLOv9	0.728	0.564	0.608	0.448	0.636

**Table 5:** Performance of YOLOv9 per every object class measured over the 2960 validation images of Use Case A.

Class	# Instances	Precision	Recall	mAP@.5	mAP@.5:.95	F1 Score
weed	1862	0.746	0.934	0.936	0.754	0.829
cable	1108	0.463	0.464	0.444	0.207	0.463
other	116	0.974	0.293	0.444	0.383	0.450

**Table 6:** YOLO performance comparison in detecting defects on TEU containers (Use Case B).

YOLO Version	Precision	Recall	mAP@.5	mAP@.5:.95	F1 Score
YOLOv4	0.622	0.882	0.717	0.428	0.730
YOLOv5	0.701	0.835	0.868	0.495	0.762
YOLOv6	0.776	0.657	0.776	0.462	0.712
YOLOv7	0.653	0.865	0.767	0.448	0.744
YOLOv8	0.706	0.731	0.727	0.496	0.718
YOLOv9	0.785	0.782	0.798	0.497	0.783

**Table 7:** YOLO performance comparison in detecting the correct or anomalous pose of AGV for automated loading in a warehouse (Use Case C).

YOLO Version	Precision	Recall	mAP@.5	mAP@.5:.95	F1 Score
YOLOv4	0.482	0.672	0.573	0.288	0.752
YOLOv5	0.743	0.784	0.813	0.558	0.850
YOLOv6	0.706	0.652	0.706	0.434	0.808
YOLOv7	0.732	0.779	0.823	0.614	0.794
YOLOv8	0.864	0.918	0.961	0.871	0.866
YOLOv9	0.866	0.863	0.922	0.838	0.849

## Data Availability

The data collected during this research work cannot be made publicly available due to confidentiality clauses of authorities, institutions, and companies involved in the projects where the research was conducted.
